# Supplementary material for: The impact of health inequity on spatial variation of COVID-19 transmission in England
Source: PLoS Comput Biol. 2024 May 28;20(5):e1012141. doi: 10.1371/journal.pcbi.1012141 (PMC11161116; doi:10.1371/journal.pcbi.1012141)
Supplement: Attachment — Submitted filename: review_pdf_version.pdf [file pcbi.1012141.s002.pdf]

Summary

The authors construct a probabilistic model that accounts for time-varying incidence across regions of England. Their model accounts for area-level risks as well as spatial connectivity between regions. They find that spatial connectivity or exportation was the primary factor driving spatial variation in risk. Shifts in dominant variant and other high-level factors (e.g. vaccine availability) were found to be key drivers of variation in incidence over time. A key goal of the study is to determine whether efforts at real-time or near real-time modeling could be improved by deeper integration of regional sociodemographic data into similar models.

Overall, this is a well thought-out, exhaustive, and technically sophisticated analysis. The authors should be commended for the thoughtfulness and thoroughness of their work. My comments and concerns outlined below should be interpreted in their intended spirit, which is to push the authors to sharpen the explanation of their results by making a more detailed comparison between their very flexible models including spatially structured and unstructured random effect terms and those relying more on locally-measured covariates. My primary concern relates to their reliance on an approach to model selection which may be inappropriate given violations of assumptions of exchangeability important to the validity of the LOO-PSIS framework, as well as conceptual difficulty in making use of their model comparison results to choose among the presented models. I will explain all of this in detail below.

Major Comments

Model Structure The parameter λ is introduced as representing the degree of undercount in the number of first infections. I think this is likely too concrete an interpretation of this parameter, as it could also be adjusting the impact of prior infections on the number of subsequent infections in a given area due to uneven or incomplete protective immunity. In all likelihood, this parameter can be best understood as a kind 'fudge factor' that is scaling the immunological lag term represented by S_{t-1} and includes some combination of the effect of prior infection on future immunity and the rate of undercounting. The authors should clarify the interpretation and role of this parameter in the model.

Robustness of findings regarding local social factors. One of the more unexpected findings of this study is that the LTLA-specific sociodemographic covariates did not have a strong association with area-level variability in risk. The authors conduct extensive sensitivity analysis to test the robustness of this finding to different model specifications. As evidenced by Table S4 in the supplement, the goodness of fit as measured by LOOIC is worst for models excluding the spatially-varying lag term (ζ_i). The differences are less dramatic for models including either a spatially varying or stationary spatial lag term (ζ).

While the authors helpfully include a sensitivity analysis showing the estimates of various social risk factors when controlled for individually vs. when all are simultaneously included in the model (Figure S60), this is only done in the presence of the spatially-varying importation term (ζ_i) and LTLA-specific random intercept (θ_i). Looking at Table S4, Model C, which includes spatially-varying ζ_i but no area-specific θ_i has a similar LOOIC value to Model A, which is the one presented in the most detail in the model. While the comparison in Figure S60 is helpful for illustrating that the correlations between these parameters do not impact their point estimates *in the presence of the spatial random effects*, the lack of detail on less-flexible models such as Model C or Models G & K makes it a bit difficult to fully assess their findings. Looking at the lower LOOIC values for models including the spatially unstructured random intercept θ_i without covariates x_i suggests that these flexible random effects are doing a quite good job of "mopping up" spatial variance and that the overall better LOOIC for the spatially flexible model may be an artifact of the choice to use the Pareto smoothed importance sampling (PSIS) along with leave-one-out cross-validation in which the information criterion is assessed one location/time combination at a time.

Chosen information criterion may be inappropriate given model assumptions and inputs. The authors state that they use the PSIS approach to computing LOO because the classic approach to LOO cross-validation is computationally expensive (i.e. leaving out one point at a time and re-running the model while computing the posterior predictive density for the held-out point). While this is true, I think it may be somewhat beside the point. The choice of pointwise LOO is unlikely to be appropriate in this setting for two reasons (for more detail, please see this document: https://mc-stan.org/loo/articles/online-only/faq.html#spatial) . First, the use of a spatial lag on the *observed* values of y_i rather than the predicted latent values, as is present in the current model, violates the assumption of conditional independence of the observations y_i necessary for LOO-based (with or without PSIS) to be valid in this setting. For spatial and time-series models (or those which are both as in this one), LOO is valid when the posterior predictive density is conditional on some latent value f_i in which the likelihood $Pr(y_i|f_i, \phi)$ is a function of the latent values (i.e. the μ_i in the current model) and parameters related to the observation process (ϕ). However, including the observed values of y_i in the spatial lag effectively marginalizes over the range of latent values, violating a key LOO assumption.

Chosen predictive information criterion should be better aligned with the scientific objectives of the modeling study. More substantively, the pointwise prediction task used to grade these models is not well-suited to the research question at hand. Specifically, the stated goal of this analysis is to improve the quality of both spatially localized and short-term predictive inference. I would argue that the appropriate predictive task to assess would be the ability of the model to predict held-out *future* values for individual locations when those locations have been held out of the model doing the prediction (i.e. under a leave-one-cluster-out scheme). This would guard against the model learning overly specific values of

the spatial lag term ζ_i which are likely to be temporally non-stationary. A LOO score obtained in this way would, to my mind, be more compelling as it would correspond more closely to the data generating process posited by the model and the intended use of this model: To integrate local contextual and spatial information to make meaningful predictions about short-term variability in risk across local jurisdictions in England. This blog post is also a helpful guide to navigating these issues:
https://statmodeling.stat.columbia.edu/2018/08/03/loo-cross-validation-approaches-valid/

The authors are right to suggest that this type of approach can be computationally expensive. However, one approach would be to chunk the data into relatively coarse spatial regions and time periods and run the model selection task on these rather than on every possible LTLA x week combination. Trading spatiotemporal resolution for conceptual clarity and operational/scientific utility is appropriate in this setting, when the higher resolution predictions may be both unstable and incorrect (due to non-exchangeability of spatiotemporal observations and the inclusion of the observed values in the spatial lag), and also conceptually difficult to interpret (e.g. with past values predicted by future ones in the locality of interest and surrounding areas).
