# Supplementary material for: The impact of health inequity on spatial variation of COVID-19 transmission in England
Source: PLoS Comput Biol. 2024 May 28;20(5):e1012141. doi: 10.1371/journal.pcbi.1012141 (PMC11161116; doi:10.1371/journal.pcbi.1012141)
Supplement: Attachment — Submitted filename: Response to reviewers.docx [file pcbi.1012141.s003.docx]

Editor’s Note:

I agree with the reviewers that this is a potentially important contribution that likely advances our understanding of health inequity on both how epidemics unfold and how it alters our perception of how they unfold. However, I also agree that a significant amount of work is needed to revise the manuscript. The authors should focus on strengthening their model comparisons, the potential bias associated with using detected cases (e.g., could the authors use the serosurveys run regularly in the England during the pandemic), and a detailed analysis of whether their information theoretic approaches to model selection are justified. The reviewers provide a detailed set of thoughtful comments, which the authors should pay careful attention to during their revision.

We thank both reviewers for their detailed and constructive comments. In responding to these comments, the most considerable revisions we have undertaken include:

- Running seven additional sensitivity analyses to explore model assumptions raised by the reviewers. These are provided in sections 4.5 – 4.8 of the supplementary material, and are referred to throughout the text with additional commentary in the main manuscript explaining these model assumptions and their context within the broader literature. None of these new sensitivity analyses changed our model outcomes or conclusions.
- We have conducted a new cross validation analysis and provided a new model comparison metric in light of reviewer 2’s helpful comments, taking their advice by designing a “leave future out” cross validation scheme that is more representative of the predictive task we are exploring. Cross validation is performed by re-fitting the model multiple times, leaving out the second half of an individual LTLA’s case data time-series in each instance. Our new model comparison metric is the combined expected log pointwise predictive density of these cross validations. This new metric has not changed the conclusions of our study.
- Following reviewer 1’s comments, we have expanded our discussion in the main manuscript of the relevant findings from community-level infection studies, and taken greater care in outlining the difference between their analyses and our own. Namely, we have stressed that our findings relate to relationships between the demographic make-up of a location and its week-to-week transmission potential, and our results should not be misconstrued as commenting on individual health outcomes.

We respond to each comment directly below. When we refer to specific lines of the text, we refer to lines in the “tracked changes” version of the manuscript.

Reviewer's Responses to Questions

Reviewer #1: In the proposed manuscript, the authors use the COVID-19 linelist data from the UK to model the SARS-CoV-2 epidemic between 2020 and 2022 at the LTLA level. They assess the importance of including LTLA-specific covariates in explaining the epidemic, concluding that ultimately, there was a limited role of factors linked to deprivation, ethnicity in driving the epidemic, while the spatial process was much more important. Overall, this represents an interesting analysis applied to very detailed series of datasets that capture the complex factors linked to SARS-CoV-2 spread. I have a few comments.

Ultimately this remains an ecological analysis comparing crude summary statistics from an area with incidence of detected cases from that area. In particular, deprivation is based on quantile – that is summarised for an area with over 100k people. There will certainly be heterogeneity within each LTLA that gets lost at this level of resolution.

The reviewer is correct to highlight the issue of heterogeneity within LTLAs for our covariates of interest. We note; however, that deprivation is not based on quantile in our model fit, but rather the continuous IMD score assigned to each LTLA as described in section 1.4 of the supplementary material. We present the mean quintile scores in Figure 1B/1D for illustrative purposes, which likely caused this misunderstanding, and we have clarified this in the caption of Figure 1. We have also changed the plot titles of Figure 1B and 1D to be clearer. While some (but not all) covariates have data available at a yet-finer spatial scale (Lower layer Super Output Areas (LSOAs)), COVID-19 case data at this level is composed of predominantly 0-counts, such that modelling is unsuitable, and the model run-time (for then 32,844 LSOAs) would prove unfeasible. Many of our variables of interest (such as time spent in different locations) are not available at finer scales than LTLA.

We have added text to the discussion (lines 348-357 in the tracked changes version) that outlines this caveat regarding heterogeneity:

*“A caveat of this study is the heterogeneity within each LTLA for some covariates of interest. LTLAs are areas of geographic administration and service provisioning, and as such differ in population sizes. While many LTLAs are close to the median LTLA population size of 142,622 people (IQR 104,869 – 237,616, see section 1.8 of the Supplementary Material), some outliers are considerably different, the largest being Birmingham with a population of 1,140,525. Heterogeneity in covariates, such as IMD, within these larger LTLAs can be observed at the Lower layer Super Output Areas (LSOAs) scale, of 32,844 regions in England, however COVID-19 case data at this scale is too sparse to model. Thus, the LTLA-scale considered demonstrates a trade-off between demographic detail, data availability, and modelling feasibility.”*

and have also added section 1.8 to the supplementary material discussing the scale of heterogeneity of some covariates, and summary statistics of the difference in population of LTLAs.

Community-based infection studies, such as the ONS and REACT studies that directly measured underlying levels of infection, provide a more direct link to individual infection risk. These community studies have tended to show a big difference in infection risk as a function of ethnicity, social deprivation, household size etc, especially early in the epidemic (see e.g., Ward et al., Nature Communications 2020). You can certainly get different conclusions when exploring the overall population level effect and individual infection risk – however, the way the paper is written, this nuance is lost. I would be careful to ensure that readers don’t conclude that there was equal infection risk by ethnicity, social deprivation, especially early in the epidemic, as this certainly wasn’t the case as shown by these direct measures. I would also include a discussion of the comparison between inferences from these community-based infection studies and this study.

We agree with the reviewer that this distinction is crucial and can, and should, be made much clearer. The paper is certainly not intended to be taken as a conclusion on individual infection risk: we do not consider denominator populations, nor do we consider clinical outcomes; rather, **we consider how the composition of a population contributes to inferred week-to-week transmission potential**. Likewise, such community-based infection studies do not directly investigate transmission or key epidemiological parameters such as the reproduction number. We have adjusted our language throughout the main text, including the title and abstract, to better stress this distinction, and we have added a paragraph to the Discussion directly highlighting the relevant community-based infection studies in the discussion (lines 285-297):

*“Crucially; however, care must be taken when comparing our results to those of community-targeted infection risk studies. Population prevalence studies, such as the REal-time Assessment of Community Transmission (REACT) studies, and those conducted via the OpenSAFELY platform, directly investigate how COVID-19 prevalence differed by demographic indicators like those considered in this study. Ward et al. (2021) identified a three-fold increase in testing antibody-positive within Black populations compared to White populations (reducing to two-fold when adjusted for confounding factors such as age, sex, IMD quintile, household size). Mathur et al. (2021) identified a similar risk for the period February 1st – August 3rd 2020, though this increased risk is not identified for the “second wave” of September 1st – December 31st 2020. Such results should not be directly compared to the findings of this study, which investigates a fundamentally different result – we do not consider denominator populations, or individual-level infection results; rather, we consider how the composition of a population contributes to week-to-week transmission potential.”*

The beta vector that links the covariates is static in time – however, some of these covariates could have changed importance over time. This should be discussed**.**

We agree that this is an important aspect to consider. To address this, we have added an additional sensitivity analysis (see section 4.5 of the supplementary material) whereby we explore fitting the model to three distinct subsections of the overall time series. May 10th 2020 - January 3rd 2021, January 10th 2021 - August 1st 2021, and, 8th 2021 - February 27th 2022.

These dates broadly correspond to, "the pre-vaccination period", "the third national lockdown" period, and the "post-lockdown" period, respectively. Figure S67 demonstrates that our results are unchanged in each instance from the main sensitivity, save for coefficients relating to time spent at home and the ASC infection control fund which, as would be expected, displayed insignificant impact during the third, latest, period - where NPIs had broadly ended.

We have added direct reference to this sensitivity analysis in the manuscript text (lines 327-331):

*“To investigate the time-specific impact of these covariates, we conducted a sensitivity analysis whereby the model was fit to three distinct subsections of the overall time series (section 4.5 of the Supplementary Material). Our results are unchanged across specific time periods, save for the significance of the ASC infection control fund disappearing for August 8th 2021 onwards, as would be expected, as this was when NPIs had been lifted.”*

The susceptibility approach makes me nervous. The model assumes an immunizing infection and that the impact of vaccination is absorbed in the zi term. However, the zi term is the other side of the equation to susceptibility. We also know that reinfection was common, especially across variants. The authors demonstrate a big effect of variant proportion on case numbers – but this is difficult to interpret in the context of assumed sterilizing immunity between variants.

To address this comment, we have added a section 4.6 to the supplementary material discussing the literature supporting this modelling decision, and further explored these assumptions with two additional sensitivity analyses.

Firstly, we refer to the UK SIREN study, assessing protection provided by primary infection amongst a cohort of health service personnel. Protection from initial infection is shown to remain strong throughout the wild-type and Alpha variant periods of the pandemic (Hall et al. (2021) doi: 10.1016/S0140-6736(21)00675-9 ), continuing throughout the Delta period, but beginning to wane for the Omicron variant (Hall et al. (2024) doi: 10.1016/j.jinf.2023.10.022). To address this issue of waning immunity, we conducted a new sensitivity analysis where we only fitted the model up to September 12^th^ 2021 – the last week before any confirmed Omicron cases are reported. Figure S68 shows that our results are not impacted by these assumptions.

The findings of the UK SIREN study are further corroborated by the systematic review conducted by Kojima et al. (2021) (doi: 10.1177/01632787211047932), which reports an average risk reduction against reinfection of 90.4% during the pre-Omicron period, and where protection against re-infection holds for, on average, up to 10 months. We conducted a second sensitivity analysis based on this specific result where we assume that a reported “first episode” only provides immunity against repeat infection for the first 10 months after it is reported.

Both these sensitivity analyses are given in section 4.6 of the supplementary material, where we show that our results are unchanged in these sensitivity analyses. We specifically refer to these inherent assumptions, and the sensitivities exploring them, in the Discussion at lines 358-365:

*“We also note a modelling assumption made whereby reported “first episodes” of disease incidence in an LTLA contributed to fully immunising protection against onwards infection. While protection against repeat infection was strong for the majority of the time period we considered, this was likely to wane more against the Omicron variant. We explored this modelling assumption through multiple sensitivity analyses where the model was fit to different time periods, and where waning of acquired immunity was assumed, which are presented in sections 4.5 and 4.6 of the Supplementary Material. Our model results were unchanged in these analyses.”*

Is there an assumption of equal reporting by covariate? So if individuals of a lower deprivation systematically reported less, would that be captured in the LTLA specific term?

This is indeed an assumption of the model. While the OpenSAFELY studies (Mathur et al. (2021) doi: 10.1016/S0140-6736(21)00634-6) identify no great disparities in testing across ethnicity for the first wave of the pandemic, they do report a slight decrease in testing within non-white ethnicity populations for the second wave. To explore this assumption, we conducted three additional sensitivity analyses.

Since the UK linelist case data reports ethnicity and IMD decile for each case, we conduct a sensitivity analysis where we double the number of Black Afr/Car cases in the data being fit to.

We additionally conduct a sensitivity analysis where we double the number of Asian-ethnicity cases in the data being fit to.

We additionally conduct a sensitivity analysis where we double the number of cases reported as being in the top two IMD deciles (the two most deprived deciles).

We present the results of these sensitivity analyses in section 4.7 of the supplementary material, where we show that our results remain unchanged by these new assumptions. We refer to these sensitivities in lines 449-450 of the text:

*“Sensitivity analyses exploring differences in reporting by ethnicity and IMD are presented in section 4.7 of the Supplementary Material.”*

It is important to note that the majority of infections were probably missed by LTLA. This study focuses just on detected cases – however many infections were subclinical or not detected via PCR (and instead by at-home antigen testing) – especially towards the end of the time series. I suspect this all gets absorbed in the zi term, however, it would be good to at least mention the role of undetected infections.

Thank you, we have specifically highlighted this additional role of the time-varying term in lines 134-137:

*“First, a time-varying random walk term, z_t, observed across all LTLAs, capturing the impact of multiple time-varying factors such as changes to non-pharmaceutical interventions (NPIs),vaccination uptake, school closures, national holidays, and changes in underlying reporting rates”*

Line 40. It would be good to get a sense of the size of LTLA (e.g., mean number of inhabitants). Is it reasonable to assume the population is homogeneous by the covariates of interest within an LTLA?

As mentioned above, we have added section 1.8 to the Supplementary Material which provides summary statistics of the distribution of LTLA populations and discusses alternate scales of data provision.

Line 67. The authors use an adjacent spatial model. I can see the reasons for doing this – however, is this the most relevant if we consider that e.g., parts of Manchester may be more connected to London than neighbouring LTLAs, and that this connectivity may change over the course of the pandemic?

A version of the model assuming a gravity-equation spatial kernel was also considered, however we neglected to provide these results in the original submission. We have now added this as an additional sensitivity analysis in section 4.8 of the Supplementary Material. This section describes the specifics of how this is modelled and demonstrates that our model results are unchanged by this alternate spatial kernel assumption. We note that this formulation incurs a (roughly) 30-fold increase in computational runtime, hence why the formulation was not chosen for the baseline analysis.

Line 73. It is worth noting that real-time modelling may have different roles at different stages of an epidemic – especially one lasting several years. The presented results are for an overall average – however, early in the epidemic, when there are many more uncertainties, the model result may look different.

Further to this point, and reflecting comments from reviewer 2, we have re-worded our references to real-time modelling throughout the paper to state our aims more clearly. Namely that such socio-demographic variables are seldom used in a real-time modelling context, but we wish to investigate if, retrospectively, with all the time afforded, these variables could be shown to have a significant impact in week-to-week transmission within local geographies. If such an effect was found, it would raise questions as to how this data could be better used in a real-time context; the reviewer rightly suggests that implementing such data under these circumstances may prove difficult. However, our study finds no such significance, supporting the decision to not prioritise their inclusion in a real-time modelling context. We also address this idea of focusing on the early period of the epidemic in the sensitivity analyses provided in section 4.5 of the supplementary material.

Figure 1B/1D – are these consistent with what was inferred from the community infection studies (ONS/REACT)? Is there a strong assumption about equal testing probability across the sub-populations and over time behind these plots?

As mentioned above, we have added text to the discussion regarding the findings of community infection studies in this context, and provided additional sensitivity analyses (section 4.7 of the supplementary material) exploring the impact of unequal test reporting by ethnicity and IMD decile.

Figure 3. I wonder again the importance of these terms at different stages of the epidemic. If the purpose of the paper is to explore a role for modelling in outbreak response, the authors may wish to present how these estimates change (if at all) at different stages of the pandemic – rather than an overall average based on data up to a time by when pretty much everyone in the country had been infected, often multiple times.

As mentioned above, this idea has been explored in a new sensitivity analysis fitting to three distinct subsections of the time series in section 4.5 of the supplementary material. Study conclusions are broadly unaffected across these sensitivities.

Figure 4. This is an interesting figure. I believe it is showing that the majority of the variance is captured by these ‘catch all’ temporal and spatial terms. Would it be possible to quantify how much of the overall variance in incidence is captured by each term? It may be that there is substantial heterogeneity at smaller spatial scales, time varying coefficients that are needed. Again – if the purpose is to understand the role of real time modelling – can these parameters be estimated reliably in an outbreak setting and what do they ultimately say?

In response to this comment we have more specifically outlined the relative scale and impact of each term comprising R_eff in lines 183-186 of the manuscript. (This was previously described in disparate sections of the supplementary material). Namely, we point out that z_t, the random walk term is of greater magnitude than the comparatively smaller LTLA-specific random-effect term theta, and of greater impact than the covariate terms. The reviewer is right to suggest that such covariates would prove challenging to include in a real-time modelling context, and our results highlight that their inclusion would not have provided informative during the real-time modelling of COVID-19.

Line 182- slightly pedantic point but diseases don’t spread - pathogens spread.

Thank you, we have corrected this.

Line 183 – is Zt correlated with vaccine uptake (it seems this data is available)? – I assume Zt covers all manner of factors driving the epidemic.

Indeed, Zt captures a variety of time-varying factors throughout the epidemic, including vaccination. We note in lines 338-347 of the discussion that due to the strong correlation between many key time-varying factors (e.g. vaccination campaigns were greatly influenced by the emergence of new variants, and NPIs were lifted following sufficient vaccinations) not all of these can be included as model variables and are instead all captured by the Zt term:

“*We have not included vaccination directly, as the vaccination rollout was itself influenced by the epidemic trajectory, with greater dose uptake encouraged in response to novel variants[22]. As such, since our model does not mechanistically include the effect of vaccination, nor the impact of waning effectiveness, the random walk term will capture both the uptake and impact of vaccination, but also unique temporal aspects such as public holidays, sporting events, seasonal patterns, and others. We see in general a reduction in the reproduction number over time, in line with the vaccine rollout, but also note increases in December likely aligned with the Christmas holidays, and other adjustments such as an increase in June/July 2021 around the time of the UEFA European Football Championship[23], followed by a drop after the event.”*

Line 232 – I’d be very careful with this statement. It is important to note that the study is modelling detected cases, not infections. If there are systematic differences by ethnicity in reporting, systematic differences in symptom risk, this would impact these estimates. In the reference cited by the authors – the paper only uses outcome measures (mortality) without considering underlying differences in the risk of infection by ethnicity – which we know was significant in the early stages of the epidemic. For example, Ward et al., Nature communications 2021 used the REACT study results to show very limited difference in mortality risk once underlying differences in infection risk were accounted for.

Thank you, the changes implemented as described in the above comments have taken care to better stress the differences between this study and community infection studies like those of Ward et al. (2021) (see lines 285-297) and to caution against direct like-for-like comparison of results. Our additional sensitivity analyses also now directly address these considerations of differences in reporting.

**Reviewer #2:**

\# Summary

The authors construct a probabilistic model that accounts for time-varying incidence across regions of England. Their model accounts for area-level risks as well as spatial connectivity between regions. They find that spatial connectivity or exportation was the primary factor driving spatial variation in risk. Shifts in dominant variant and other high-level factors (e.g. vaccine availability) were found to be key drivers of variation in incidence over time. A key goal of the study is to determine whether efforts at real-time or near real-time modeling could be improved by deeper integration of regional sociodemographic data into similar models.

Overall, this is a well thought-out, exhaustive, and technically sophisticated analysis. The authors should be commended for the thoughtfulness and thoroughness of their work. My comments and concerns outlined below should be interpreted in their intended spirit, which is to push the authors to sharpen the explanation of their results by making a more detailed comparison between their very flexible models including spatially structured and unstructured random effect terms and those relying more on locally-measured covariates. My primary concern relates to their reliance on an approach to model selection which may be inappropriate given violations of assumptions of exchangeability important to the validity of the LOO-PSIS framework, as well as conceptual difficulty in making use of their model comparison results to choose among the presented models. I will explain all of this in detail below.

Thank you, we address these points specifically below.

\# Major Comments

**Model Structure** The parameter $\lambda$ is introduced as representing the degree of undercount in the number of first infections. I think this is likely too concrete an interpretation of this parameter, as it could also be adjusting the impact of prior infections on the number of subsequent infections in a given area due to uneven or incomplete protective immunity. In all likelihood, this parameter can be best understood as a kind 'fudge factor' that is scaling the immunological lag term represented by $S_{t-1}$ and includes some combination of the effect of prior infection on future immunity and the rate of undercounting. The authors should clarify the interpretation and role of this parameter in the model.

We agree with the reviewer that our initial description was overly simplistic and have adjusted the Methods section to better reflect the broader array of factors that the lambda term is capturing (lines 414-417):

*“…and λ is a scaling factor parameter, between 0 and 1, scaling the acquired-immunity lag term to account for the impact of under-reporting of first episodes, incomplete protection of acquired immunity, and other nationwide scaling effects”*

**Robustness of findings regarding local social factors.** One of the more unexpected findings of this study is that the LTLA-specific sociodemographic covariates did not have a strong association with area-level variability in risk. The authors conduct extensive sensitivity analysis to test the robustness of this finding to different model specifications. As evidenced by Table S4 in the supplement, the goodness of fit as measured by LOOIC is worst for models excluding the spatially-varying lag term ($\zeta_i$). The differences are less dramatic for models including either a spatially varying or stationary spatial lag term ($\zeta$).

While the authors helpfully include a sensitivity analysis showing the estimates of various social risk factors when controlled for individually vs. when all are simultaneously included in the model (Figure S60), this is only done in the presence of the spatially-varying importation term ($\zeta_i$) and LTLA-specific random intercept ($\theta_i$). Looking at Table S4, Model C, which includes spatially-varying $\zeta_i$ but no area-specific $\theta_i$ has a similar LOOIC value to Model A, which is the one presented in the most detail in the model. While the comparison in Figure S60 is helpful for illustrating that the correlations between these parameters do not impact their point estimates *in the presence of the spatial random effects*, the lack of detail on less-flexible models such as Model C or Models G & K makes it a bit difficult to fully assess their findings. Looking at the lower LOOIC values for models including the spatially unstructured random intercept $\theta_i$ without covariates $x_i$ suggests that these flexible random effects are doing a quite good job of "mopping up" spatial variance and that the overall better LOOIC for the spatially flexible model may be an artifact of the choice to use the Pareto smoothed importance sampling (PSIS) along with leave-one-out cross-validation in which the information criterion is assessed one location/time combination at a time.

We agree with the reviewer that such analyses would prove a useful addition for model comparison purposes, and as such have recreated plot S60 (now plot S61 in the updated supplementary material) for models C, G, and K – figs S62, S63, and S64 respectively. In general, this reduction of flexibility results in, as expected, smaller width of confidence intervals for the respective beta coefficients, better highlighting the impact of correlation between variables. We also see for the especially restrictive models G and K, that our beta coefficients shrink closer to 0, as would be expected as a less flexible characterisation of attacking cases is provided.

****Chosen information criterion may be inappropriate given model assumptions and inputs**.** The authors state that they use the PSIS approach to computing LOO because the classic approach to LOO cross-validation is computationally expensive (i.e. leaving out one point at a time and re-running the model while computing the posterior predictive density for the held-out point). While this is true, I think it may be somewhat beside the point. The choice of pointwise LOO is unlikely to be appropriate in this setting for two reasons (for more detail, please see this document: https://mc-stan.org/loo/articles/online-only/faq.html#spatial) . First, the use of a spatial lag on the *observed* values of $y_i$ rather than the predicted latent values, as is present in the current model, violates the assumption of conditional independence of the observations $y_i$ necessary for LOO-based (with or without PSIS) to be valid in this setting. For spatial and time-series models (or those which are both as in this one), LOO is valid when the posterior predictive density is conditional on some latent value $f_i$ in which the likelihood $Pr(y_i |f_i, \phi )$ is a function of the latent values (i.e. the $\mu_i$ in the current model) and parameters related to the observation process ($\phi$). However, including the observed values of $y_i$ in the spatial lag effectively marginalizes over the range of latent values, violating a key LOO assumption.

****Chosen predictive information criterion should be better aligned with the scientific objectives of the modeling study**.** More substantively, **the pointwise prediction task used to grade these models is not well-suited to the research question at hand**. Specifically, the stated goal of this analysis is to improve the quality of both spatially localized and short-term predictive inference. I would argue that the appropriate predictive task to assess would be the ability of the model to predict held-out *future* values for individual locations when those locations have been held out of the model doing the prediction (i.e. under a leave-one-cluster-out scheme). This would guard against the model learning overly specific values of the spatial lag term $\zeta_i$ which are likely to be temporally non-stationary. A LOO score obtained in this way would, to my mind, be more compelling as it would correspond more closely to the data generating process posited by the model and the intended use of this model: To integrate local contextual and spatial information to make meaningful predictions about short-term variability in risk across local jurisdictions in England. This blog post is also a helpful guide to navigating these issues: https://statmodeling.stat.columbia.edu/2018/08/03/loo-cross-validation-approaches-valid/.

The authors are right to suggest that this type of approach can be computationally expensive. However, one approach would be to chunk the data into relatively coarse spatial regions and time periods and run the model selection task on these rather than on every possible LTLA x week combination. Trading spatiotemporal resolution for conceptual clarity and operational/scientific utility is appropriate in this setting, when the higher resolution predictions may be both unstable and incorrect (due to non-exchangeability of spatiotemporal observations and the inclusion of the observed values in the spatial lag), and also conceptually difficult to interpret (e.g. with past values predicted by future ones in the locality of interest and surrounding areas).

We greatly appreciate the reviewer’s time in providing a detailed assessment and suggestion of a more suitable measure of model comparison. On reflection of the materials provided by the reviewer we agree that LOOIC is likely a flawed metric in this particular circumstance, and have followed the reviewer’s suggestion of instead providing a new measure of model comparison that better reflects the predictive task being considered. Our new measure may generally be considered as the expected log pointwise predictive density (elpd) of a “Leave future out” (LFO) cross-validation scheme. This has replaced the previous LOOIC in both the main manuscript and section 4.2 of the supplementary material. We detail this calculation below.

For each of the twelve models, we fit the model 306 times, each time leaving out of the fitting process the final 48 weeks of the time-series for one particular LTLA, and in each instance calculating the posterior log-likelihood for the data that was not fit to. These 306 log-likelihoods are then summed together to create a final elpd (LFO) score for each model, with a greater score (lower magnitude) indicating a better fit. As you will see, the key findings remain unchanged - that the inclusion of the beta covariates is of insignificant impact to model fit when compared with the impact of including spatial importation.

Comparing models C,G,K via this new metric requires more careful consideration, as the models including the theta error term will be fitting LTLA-specific error parameters, theta_i, and importation parameters, zeta_i, to only the first half of their time series. As such, it makes sense for the mean elpd_LFO score to be greater for model C than model A, as it is effectively not being “overfit” by overly specific error terms theta_i.

We have retained our initial LOOIC metrics within the supplementary material (moved to a new section, 4.9) but we specifically highlight the caution that should be taken when interpreting these results, as the reviewer pointed out.
