# Supplementary material for: The impact of health inequity on spatial variation of COVID-19 transmission in England
Source: PLoS Comput Biol. 2024 May 28;20(5):e1012141. doi: 10.1371/journal.pcbi.1012141 (PMC11161116; doi:10.1371/journal.pcbi.1012141)
Supplement: Attachment — Submitted filename: Response to reviewers.docx [file pcbi.1012141.s004.docx]

We thank the editors and reviewers for their prompt responses. Below we respond directly to each of the additional comments from reviewer 3, which we have addressed through alterations and additions to the text. When referring to specific line numbers, we are referring to the “tracked changes” version of the manuscript.

Comments to the Authors:

Reviewer #1: The authors have done an excellent job of responding to my concerns. I have no further comments.

We thank the reviewers again for their valuable and constructive comments.

Reviewer #3: Reviewer 1 brought up several crucial points, many of which resonated with my own observations. I was impressed to see the revisions and the authors' thoroughness in conducting additional sensitivity analyses in great detail.

I have a couple of minor comments, along with one concern regarding the clarity of the results' interpretation (point 5):

1. How is statistical significance of variables defined?

When we refer to “statistical significance” within the manuscript we are referring to the 95% CI approach. A variable is referred to as “statistically significant” if the 95% CI of its beta coefficient posterior distribution does not span 0. We have now explicitly stated this in the manuscript at lines 424-426:

*“A covariate is considered statistically significant if the 95% CI of its β coefficient posterior distribution does not span 0.”*

2. Administrative regions or areas for LTLAs- needs consistency in the manuscript, including the title. <https://www.ons.gov.uk/methodology/geography/ukgeographies/administrativegeography/england>

The reviewer is right to highlight that the general term “region” could be confused with the precise English geographies named “regions” if usage is not consistent. To address this, we have changed reference to “regions” throughout the text to “administrative areas”.

3. The authors mention mechanistic impact in the abstract. How is this defined? I didn’t see it anywhere in the main manuscript or the supplementary methods.

The use of the term “mechanistic” here was intended to refer to how the inclusion of covariates is mechanistically (how the model is written/structured) distinct from how spatial importation is considered. We have now removed the term “mechanistic”, referring only to “impact” to avoid confusion.

4. In addition to Reviewer 1’s comment and the authors’ response to it “Line 67. The authors use an adjacent spatial model. I can see the reasons for doing this – however, is this the most relevant if we consider that e.g., parts of Manchester may be more connected to London than neighbouring LTLAs, and that this connectivity may change over the course of the pandemic?” I was hoping that the authors would consider mobility patterns to quantify connectivity, rather than Euclidean distances and a gravity model. I think it would be a stronger predictor if such a variable were to be used in real time. Or making a note of this in the discussion.

The reviewer is right that our adjacency-based spatial kernel is a simplification of reality. Studies that used mobility pattern data from mobile phone networks (see Kraemer et al. (2021) ref [26]) did so using privately acquired data that we did not have access to. We have now addressed this explicitly in the discussion at lines 368-369:

*“The notable impact of our simplified adjacency kernel would likely be strengthened further were it supplemented with such direct measures of individual mobility patterns.”*

5. The last sentence in the abstract says ”While these results confirm the impact of some, but not all, measures of regional inequity in England, our work corroborates the finding that observed differences in regional disease transmission during the pandemic were predominantly driven by underlying epidemiological factors rather than the demography and health inequity between regions.” I think it is important to think about why the important drivers i.e. epidemiological parameters, mobility patterns could be different between areas. I would argue that inequity is a complex phenomenon (both socially and as a quantitative measure) and is probably a crucial underlying cause of the differences in epidemiological factors. This phrasing makes me nervous about its interpretation by readers. I would request the authors to be more specific in their concluding statement in the abstract which can be easily misinterpreted. It would be more appropriate and transparent to say that the static, aggregated sociodemographic variables don’t add much value in prediction of transmission or effective reproduction number. And this work would motivate the field to move forward in a direction where we try to understand the underlying causes and drivers of the epidemiological factors which are in turn the drivers of transmission. But from the point of view of prediction in real time and improving model fit (as described in the last section of the discussion), I agree that these poorly collected, outdated and often incomplete measures of inequity don’t add much value, as they currently stand. And I would be more confident with a similar careful phrasing in the abstract.

We thank the reviewer for this important point. We agree that more care should be taken to avoid presenting such a harsh distinction between inequity and epidemiological factors. We have re-worded the abstract, while trying to maintain a succinct summary, and have added further discussion on this point in lines 370-377:

*“It is important to note that the highlighted impact of epidemiological factors, such as mobility patterns and contact networks, does not exclude the importance of health inequity in pandemic response. Health inequity is a complex phenomenon that extends beyond aggregated measures of deprivation and access-to-care, and further work is required to investigate its relation, both socially and quantitatively, to these underlying epidemiological factors. Our results should not be interpreted as ruling out the impact of health inequity in shaping epidemic trajectories; rather, that the aggregated measures considered do not directly improve model prediction of week-to-week transmission within a geography.”*

6. Is it possible that the random walk term captures many of the factors that constitute inequity and as expected is one of the greatest contributors to Reff?

While the random walk term is indeed a substantial contributor to Reff, this is likely better interpreted as the impact of NPIs and vaccination. The random walk term acts equally on all LTLAs (does not vary for each LTLA), and so only captures factors that are affecting transmission across the whole country simultaneously, and thus not capturing differences in provisioning to different parts (and populations) of the country.

The spatial error term, theta, however, does capture unexplained underlying differences across LTLAs that could represent inequities not captured by the covariates considered, as mentioned in lines 204-206:
*“The apparent clustering of higher and lower theta_i values suggests this pattern may not simply be noise, but that there could be spatial variables yet to be identified that may also be influencing the transmission of COVID-19 across LTLAs in England.”*

7. The authors have made the distinction between detected cases and infections and provided sensitivity analysis for a few different reporting scenarios. Reporting (for lateral flow tests: sensitivity analysis 4.4 was done to include data from UK COVID-19 data dashboard) and test seeking behaviour (PCR which is the primary data used) likely changed over time and by the different groups. In addition to the analyses, it would be good make it clear that the Pillar 2 PCR dataset is likely a combination of the true (primarily symptomatic) cases, test seeking behaviour and changes in intended use of the tests (for symptomatic testing, confirmatory tests)– around line 78 or 287.

We have added mentioned of this to line 391 where reasoning for using Pillar 2 PCR cases is discussed.
